# Convergence of Cerebrospinal Fluid Neuronal Biomarker Burden and MRI Lesion Pattern and Its Association With Short‐Term Outcome in Neuroinfectious Diseases

**DOI:** 10.1111/ene.70802

**Published:** 2026-11-02

**Authors:** Lucio D'Anna, Deborah K. Erhart, Franz Felix Konen, Christopher M. Weise, Makbule Senel, Caroline Stapf, Dominica Hudasch, Petra Steinacker, Lorenzo Barba, Patrick Oeckl, Jonas Ranke, Nicola Ticozzi, Federico Verde, Steffen Halbgebauer, Thomas Skripuletz, Kurt‐Wolfram Sühs, Hayrettin Tumani, Markus Otto, Samir Abu‐Rumeileh

**Affiliations:** ^1^ Department of Brain Sciences Imperial College London London England UK; ^2^ Department of Stroke and Neuroscience Charing Cross Hospital, Imperial College London Healthcare NHS Trust London England UK; ^3^ Department of Neurology Ulm University Hospital Ulm Germany; ^4^ Department of Neurology Hannover Medical School Hannover Germany; ^5^ Department of Neurology Martin‐Luther‐University Halle‐Wittenberg Halle (Saale) Germany; ^6^ German Center for Neurodegenerative Diseases (DZNE e.V.) Ulm Germany; ^7^ Department of Neurology and Laboratory of Neuroscience IRCCS Istituto Auxologico Italiano Milan Italy; ^8^ Department of Pathophysiology and Transplantation, “Dino Ferrari” Center Università Degli Studi di Milano Milan Italy; ^9^ Cluster of Excellence RESIST (EXC 2155) Hannover Medical School Hannover Germany

**Keywords:** biomarkers, central nervous system infections, cerebrospinal fluid, magnetic resonance imaging, prognosis

## Abstract

**Background:**

Neuroinfectious diseases (NDs) are potentially treatable conditions associated with substantial morbidity, but their clinical course remains difficult to predict. While specific MRI patterns are characteristic of distinct aetiologies, their prognostic value remains poorly investigated. Cerebrospinal fluid (CSF) biomarkers of neuroaxonal and synaptic damage have been linked to clinical outcome. We investigated the association of CSF biomarkers and MRI findings with short‐term outcome in NDs.

**Methods:**

In this multicentre cohort study, CSF was analysed in 123 patients with NDs and in 95 non‐neuroinflammatory controls. Concentrations of beta‐synuclein, SNAP‐25, neurogranin and neurofilament light chain protein (NfL) were measured. MRI findings were classified by topography and lesion pattern. Clinical outcome at discharge was assessed using the modified Rankin Scale and degree of recovery.

**Results:**

All CSF biomarkers were higher in ND patients with MRI abnormalities than in those without MRI abnormalities and/or controls, also within the (meningo‐)encephalitis group. Temporal lobe involvement was the MRI pattern most consistently associated with elevated CSF biomarkers, even after multivariable adjustment including aetiology, and was independently associated with poor functional outcome and incomplete recovery. A global neuronal biomarker burden derived by principal component analysis (PC1) improved the fit of outcome models including MRI lesion pattern, with both temporal lobe involvement and PC1 remaining independently associated with outcome at discharge.

**Conclusions:**

MRI lesion pattern and CSF biomarkers of synaptic and neuroaxonal damage were independently associated with short‐term outcome in NDs, suggesting that they capture complementary aspects of disease severity. Prospective studies are needed to establish their prognostic value.

## Graphical abstract

In 123 patients with neuroinfectious diseases, temporal lobe involvement on MRI was associated with higher CSF neuronal biomarkers. Both temporal lobe involvement and global CSF neuronal biomarker burden were independently associated with outcome at discharge. MRI and CSF biomarkers thus provide complementary aspects of disease severity. Created in Biorender. Abu Rumeileh, S. (2026) https://BioRender.com/5jso9cp.

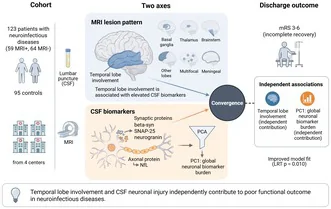

## 1 Introduction

Neuroinfectious diseases (NDs) represent a heterogeneous group of conditions encompassing viral, bacterial and other infectious aetiologies, manifesting with meningitis, (meningo‐)encephalitis, myelitis, cranial nerve involvement and other clinical presentations [1, 2]. Despite being potentially treatable, NDs are associated with substantial short‐ and long‐term morbidity and mortality. Indeed, up to 30%–50% of patients experience persistent neurological sequelae even after appropriate therapy, yet the clinical course remains difficult to predict at the individual level [1, 2, 3].

Magnetic resonance imaging (MRI) and cerebrospinal fluid (CSF) analysis represent complementary pillars in the evaluation of NDs, capturing different aspects of nervous system involvement [1, 2, 3, 4]. While MRI provides information on the topography and extent of brain lesions, CSF routine parameters and microbiological investigations reflect biological processes as well as the underlying aetiology [1, 2, 3]. In detail, specific MRI patterns have been associated with distinct infectious aetiologies; most notably, temporal lobe signal abnormalities are a hallmark of herpes simplex virus (HSV) encephalitis [3, 5]. In contrast, tick‐borne encephalitis (TBE) more frequently involves the thalamus and brainstem, while bacterial meningitis may produce leptomeningeal enhancement [3, 5]. However, the prognostic value of MRI findings in NDs has been far less systematically investigated than their diagnostic utility. While MRI abnormalities have been independently associated with poor outcome in HSV, TBE, central nervous system (CNS) tuberculosis and bacterial meningoencephalitis [3, 4, 6, 7], evidence for other ND aetiologies remains inconsistent [3, 4, 6]. Similarly, the prognostic value of CSF routine parameters and microbiological investigations is variable across studies [1, 2].

In contrast, CSF biomarkers reflecting downstream pathophysiological mechanisms beyond neuroinflammation, such as neuroaxonal damage (e.g., neurofilament light chain protein, NfL) and synaptic injury (e.g., neurogranin; synaptosomal‐associated protein 25, SNAP‐25; beta‐synuclein, beta‐syn), have emerged as promising indicators of clinical outcome in NDs [8, 9, 10]. Our previous multicentre study showed that these markers are increased in patients with NDs and that their concentrations track both with the severity of the clinical picture and with functional recovery at discharge [8]. Notably, higher levels of CSF biomarkers were observed in patients with inflammatory changes on neuroimaging, suggesting a potential link between MRI‐defined lesion patterns and the degree of underlying neuroaxonal and synaptic damage [8].

However, studies combining MRI lesion patterns and CSF biomarkers of neuronal and synaptic damage in relation to clinical outcome are lacking. In this multicentre cohort of patients with diverse ND aetiologies, we investigated the relationship between MRI lesion topography and CSF biomarker profiles and their individual and combined association with short‐term outcome.

## 2 Methods

### 2.1 Study Design and Participants

This retrospective multicentre study, which drew on four European Neurology Departments, namely the Universities of Halle (Saale), Ulm, Hannover and Milan, comprised 123 patients with NDs and 95 non‐neuroinflammatory controls, all of whom had already been enrolled in our previous work [8]. Eligible ND patients had a clinical picture of meningitis and/or (meningo‐)encephalitis, myelitis, polyradiculitis, vasculitis or cranial nerve involvement or any combination of these and were required to show CSF pleocytosis, defined as a white blood cell count > 5 cells/μL [8, 11]. The diagnosis of encephalitis followed the International Encephalitis Consortium criteria [12]. Additional information regarding inclusion criteria and specific ND aetiologies is reported elsewhere [8] and in the supplementary methods. Non‐neuroinflammatory controls (*n* = 95) were cases without clinical, radiological or CSF findings suggestive of an inflammatory or neurodegenerative neurological disease [8] (supplementary methods).

Reporting followed the STROBE recommendations for observational research and the study complied with Good Clinical Practice standards and with the Declaration of Helsinki in its revised form. The local ethics committees of the four participating centres granted approval: Ulm University Hospital (20/10), Hannover Medical School (7837_BO_K_2018), Martin‐Luther‐University Halle‐Wittenberg (2021–101) and IRCCS Istituto Auxologico Italiano, Milan (2021_05_18_04). Written informed consent was obtained from all participants included in the study or their legal guardians.

### 2.2 Clinical, Neuroimaging and Biomarker Data

The following variables were retrieved for ND patients whenever documented: presenting clinical syndrome; disease duration, namely the interval between symptom onset and lumbar puncture (LP); routine CSF parameters (pleocytosis and oligoclonal bands); brain and/or spinal cord MRI findings; electroencephalogram data; time from symptom onset to MRI; functional status on the modified Rankin Scale (mRS) at LP and at discharge; need for intensive care unit admission; and length of hospital stay [8]. Outcome at discharge was operationalised either as the degree of recovery (complete versus incomplete) or as the mRS value at that time point, with scores in the 0–2 range denoting a good and scores in the 3–6 range a poor outcome [8].

Brain and spinal cord MRI were performed according to standard clinical protocols and evaluated for the presence and topography of signal abnormalities on T2/FLAIR and post‐contrast T1‐weighted sequences and, if available, other sequences. MRI findings were classified as normal or abnormal and the topography of lesions was recorded by brain region involvement (unilateral or bilateral temporal, unilateral or bilateral non‐temporal lobe, basal ganglia/thalamus, brainstem, cranial and/or peripheral nerves, spinal cord) and pattern (multifocal lesions with white matter involvement, multifocal lesions with grey and white matter involvement, intra‐axial space‐occupying and/or ring‐enhancing lesion, leptomeningeal contrast enhancement, extra‐axial lesion). MRI data were reviewed and classified by three investigators (SAR, DKE, FFK), each at their respective centre, based on official neuroradiology reports and a pre‐specified taxonomy adapted from previously published frameworks [12, 13, 14, 15, 16]. Formal inter‐rater reliability assessment was therefore not feasible given the multicentre design.

CSF samples were collected by lumbar puncture performed during the same hospitalisation as MRI. CSF biomarkers beta‐syn, SNAP‐25, neurogranin and NfL were measured with an in‐house immunoassay and commercial immunoassays as previously described [8] (Supplementary methods).

### 2.3 Statistical Analysis

Statistical analyses were performed using R (version 4.4.1). Continuous variables were summarised as median and interquartile range (IQR) and compared using the Kruskal–Wallis test. For CSF biomarkers, when overall group differences were significant, pairwise comparisons were performed as post hoc analyses using the Wilcoxon rank‐sum test with Benjamini–Hochberg correction for multiple testing. Categorical variables were expressed as counts and percentages and compared using the *χ* ^2^ test or Fisher's exact test, as appropriate.

To evaluate differences in CSF biomarker levels across diagnostic groups and MRI lesion patterns, univariable analyses were performed, followed by multivariable linear regression models. Each MRI feature was modelled separately, adjusting for age, sex, time to LP and diagnostic aetiology (HSV vs. other aetiologies). Biomarker values were standardised (*z*‐scores) prior to modelling to allow comparison of effect sizes across markers with different measurement scales. Multivariable models were additionally repeated after exclusion of patients with HSV infection.

To integrate markers of synaptic and neuroaxonal injury into a single biological construct, principal component analysis (PCA) was performed using the synaptic composite score (including beta‐syn, neurogranin and SNAP‐25) and log‐transformed NfL values. The first principal component (PC1) was retained as a summary measure of global neuronal biomarker burden, representing a shared dimension of neuronal injury. The discriminatory performance of PC1 for different MRI patterns was assessed using receiver operating characteristic (ROC) curve analysis. Logistic regression models were fitted using PC1 as the predictor, both unadjusted and adjusted for age, sex and time to lumbar puncture. Model performance was quantified using the area under the curve (AUC) and comparisons between models were performed using DeLong's test. Associations between MRI features and clinical outcomes at discharge were evaluated using multivariable logistic regression models adjusted for age, sex, time to LP, diagnostic aetiology and clinical severity (mRS at the time of CSF sampling). Additional models were constructed including PC1 to assess whether the global biomarker burden provided independent information beyond MRI features. Model fit was compared using likelihood ratio tests and the Akaike information criterion (AIC). Sensitivity analyses were performed in patients with (meningo‐)encephalitis (MRI+ vs. MRI−) and, to address confounding by HSV aetiology, in patients with temporal involvement (HSV vs. non‐HSV) and in non‐HSV patients (with vs. without temporal involvement). All comparisons were performed using the Wilcoxon rank‐sum test. A two‐sided *p* < 0.05 was considered statistically significant.

## 3 Results

### 3.1 CSF Biomarker Distribution Across Diagnostic Groups

Baseline demographic characteristics and CSF biomarker levels across diagnostic groups are summarised in Table S1. Additional demographic, clinical and paraclinical features of the ND group are reported in Table S2. The cohort comprised 64 patients with NDs without MRI abnormalities (ND‐MRI−) and 59 patients with NDs with MRI abnormalities (ND‐MRI+) as well as 95 controls. Patients with ND‐MRI+ were significantly older compared with both ND‐MRI− patients and controls (*p* = 0.001), while sex distribution did not differ significantly across groups (*p* = 0.115). The interval between symptom onset and MRI did not differ between ND‐MRI− and ND‐MRI+ patients (*p* = 0.52) (Table S2).

Global differences across diagnostic groups were observed for all CSF biomarkers (beta‐syn and NfL *p* < 0.001, neurogranin *p* = 0.002, SNAP‐25 *p* = 0.014) (Figure 1, Table S1). Beta‐syn and NfL concentrations differed significantly across all three groups, with the highest levels observed in ND‐MRI+ patients, intermediate levels in ND‐MRI− patients and the lowest levels in controls. All pairwise comparisons were significant for both biomarkers (beta‐syn: *p* = 0.013, *p* < 0.001 and *p* < 0.001; NfL: *p* = 0.005, *p* < 0.001 and *p* < 0.001 for ND‐MRI− vs. controls, ND‐MRI+ vs. controls and ND‐MRI+ vs. ND‐MRI−, respectively). Neurogranin levels were significantly higher in ND‐MRI+ patients compared with both ND‐MRI− patients and controls (*p* = 0.024 and *p* = 0.002, respectively), while no difference was observed between ND‐MRI− patients and controls (*p* = 0.393). In contrast, SNAP‐25 levels differed significantly only between ND‐MRI+ and ND‐MRI− patients (*p* = 0.018), while no significant differences were observed between controls and ND‐MRI− (*p* = 0.101) or between controls and ND‐MRI+ patients (*p* = 0.105) (Figure 1).

**FIGURE 1:**
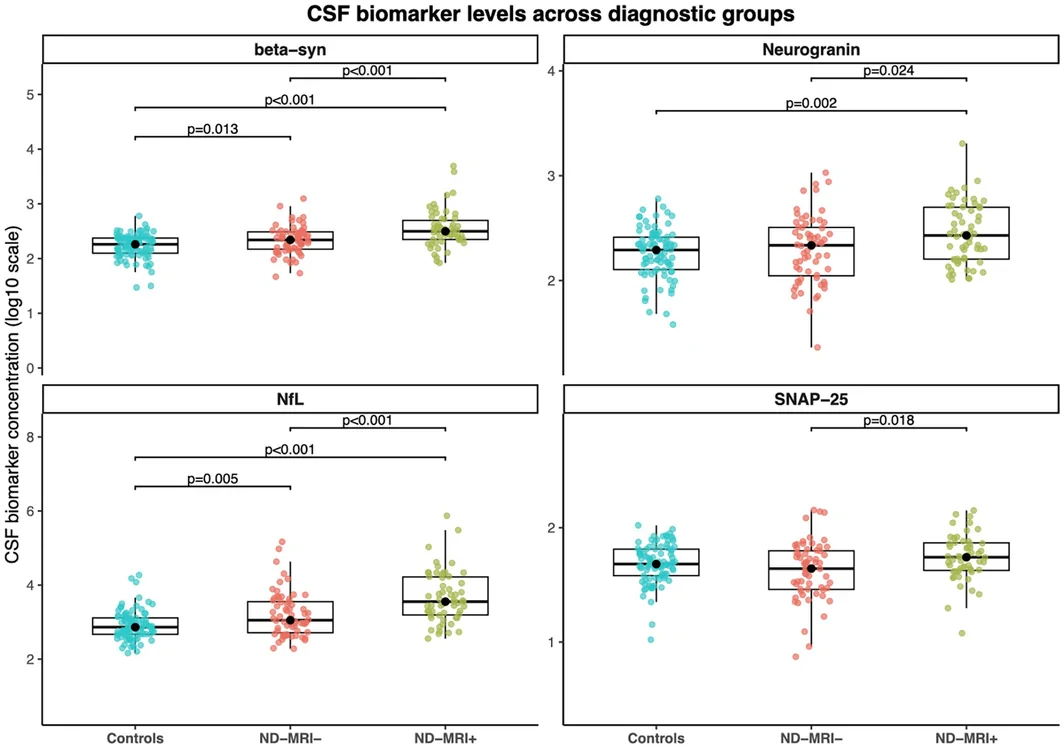
CSF biomarker levels across diagnostic groups. CSF concentrations of beta‐syn, neurogranin, NfL and SNAP‐25 in non‐neuroinflammatory controls and in patients with NDs without (ND‐MRI−) and with (ND‐MRI+) MRI abnormalities. Concentrations (pg/mL) are displayed on a log10 scale. Box plots indicate medians and interquartile ranges and coloured dots represent individual values. Only significant pairwise comparisons (Wilcoxon rank‐sum test, Benjamini–Hochberg‐adjusted) are shown. Beta‐syn, beta‐synuclein; NDs, neuroinfectious diseases; NfL, neurofilament light chain protein; SNAP‐25, synaptosomal‐associated protein 25.

In a sensitivity analysis restricted to patients with (meningo‐)encephalitis (*n* = 85; MRI+‐, *n* = 47; MRI, *n* = 38), biomarker concentrations remained significantly higher in MRI+ patients (beta‐syn *p* = 0.011, neurogranin *p* = 0.017, SNAP‐25 *p* = 0.016, NfL *p* = 0.037) (Table S3).

### 3.2 Associations Between MRI Patterns and CSF Biomarker Profiles and Their Discriminatory Performance

CSF biomarker levels according to specific MRI features are reported in Table 1. Here, we restricted the analyses to MRI pattern subgroups with sufficient sample size and combined selected categories (temporal lobe involvment, other lobe involvement, basal ganglia/thalamus and/or brainstem involvement, multifocal grey and/or white matter lesions and meningeal contrast enhancement). Temporal lobe involvement was associated with significantly higher concentrations of all investigated biomarkers. In particular, patients with temporal lobe abnormalities showed increased levels of beta‐syn (median 384.7 vs. 240.8 pg/mL, *p* = 0.002), SNAP‐25 (66.4 vs. 46 pg/mL, *p* < 0.001), neurogranin (411.2 vs. 209.5 pg/mL, *p* < 0.001) and NfL (4181 vs. 1816.5 pg/mL, *p* = 0.004) compared with those without temporal involvement.

**TABLE 1:** CSF biomarker distribution according to MRI features.

	Biomarker (pg/mL) median (IQR)	No	Yes	*p*
Temporal lobe involvement	CSF beta‐syn	240.8 (156.8–328)	384.7 (277–657.3)	0.002
	CSF SNAP‐25	46 (29.6–61.6)	66.4 (54.4–82)	< 0.001
	CSF neurogranin	209.5 (124.7–325.6)	411.2 (278.7–545)	< 0.001
	CSF NfL	1816.5 (700–4945)	4181 (2745–17,145)	0.004
Basal ganglia/thalamus and/or brainstem involvement	CSF beta‐syn	256 (161.3–348.1)	424 (355.8–549.7)	0.046
	CSF SNAP‐25	48.5 (32.3–67.3)	51.4 (50.4–53.9)	0.438
	CSF neurogranin	222 (128.8–374.2)	326.5 (210.5–462.5)	0.181
	CSF NfL	1958.5 (786.8–5001.5)	15,893 (6384–21,487)	0.008
Other lobe involvement (non‐temporal)	CSF beta‐syn	261.5 (161.3–367.3)	264.2 (225–346.2)	0.804
	CSF SNAP‐25	47.9 (32.1–66.8)	60.4 (53–71)	0.106
	CSF neurogranin	225.4 (128.8–374.2)	163.9 (160.1–502.2)	0.745
	CSF NfL	1958.5 (786.8–5498.5)	3821 (2816–6413)	0.191
Multifocal lesions with grey and/or white matter involvement	CSF beta‐syn	253.3 (171.4–355.8)	332 (256.1–508.5)	0.183
	CSF SNAP‐25	48.3 (32.2–67)	52.9 (42.7–76.9)	0.273
	CSF neurogranin	218.2 (127.5–359.4)	363.6 (191.7–505.7)	0.091
	CSF NfL	1985 (780–5448)	4608 (1854.2–18,924.5)	0.106
Meningeal contrast enhancement	CSF beta‐syn	252.4 (156.8–367.3)	317.8 (256.1–348.7)	0.152
	CSF SNAP‐25	49 (31.5–67.4)	43.2 (40.1–65.1)	0.993
	CSF neurogranin	216.3 (124.8–358.8)	358.8 (253.7–437)	0.064
	CSF NfL	2003 (825–5516.5)	2797 (859–3308)	0.944

*Note:* Values are median (IQR) in pg/mL. *p* values refer to the comparison between patients with and without each MRI feature (Wilcoxon rank‐sum test). MRI categories were combined where necessary to obtain subgroups of sufficient size.

Abbreviations: beta‐syn, beta‐synuclein; IQR, interquartile range; NfL, neurofilament light chain protein; SNAP‐25, synaptosomal‐associated protein 25.

Temporal lobe involvement was observed in 11 patients with HSV and in 10 with other aetiologies (bacterial meningoencephalitis, *n* = 6; varicella zoster virus, *n* = 4), whereas 9 of the 20 HSV patients showed no temporal lobe involvement. Biomarker concentrations in non‐HSV patients with temporal lobe involvement were comparable to those in HSV patients with temporal lobe involvement (all *p* > 0.5) and higher than in non‐HSV patients without temporal lobe involvement (beta‐syn *p* = 0.002, neurogranin *p* = 0.006, SNAP‐25 *p* = 0.004, NfL *p* = 0.030) (Table S4).

Basal ganglia/thalamus and/or brainstem involvement was associated with higher beta‐syn and NfL levels, reaching statistical significance for both biomarkers (*p* = 0.046 and *p* = 0.008, respectively), whereas no significant differences were observed for SNAP‐25 or neurogranin (Table 1). In contrast, other MRI patterns, including non‐temporal lobe involvement, multifocal lesions and meningeal contrast enhancement, were not consistently associated with CSF biomarker levels (Table 1).

These findings were largely confirmed in separate multivariable linear regression analyses, with each MRI pattern modelled individually and adjustment for age, sex, time to LP and diagnostic aetiology (HSV vs. other aetiologies) (Table S5). In the whole cohort, temporal lobe involvement remained independently associated with higher levels of all CSF biomarkers, including beta‐syn (*β* = 1.07, 95% CI 0.48–1.66; *p* < 0.001), neurogranin (*β* = 0.92, 95% CI 0.37–1.48; *p* = 0.001), SNAP‐25 (*β* = 1.08, 95% CI 0.51–1.64; *p* < 0.001) and NfL (*β* = 0.90, 95% CI 0.28–1.52; *p* = 0.005) (Table 2). In multivariable models restricted to non‐HSV patients (*n* = 103), temporal lobe involvement remained significantly associated with higher beta‐syn and neurogranin levels, while the association with SNAP‐25 showed a trend toward significance and that with NfL was no longer significant, possibly reflecting the limited statistical power of this analysis (Table S6).

**TABLE 2:** Adjusted associations between MRI patterns and CSF biomarkers.

MRI pattern	Biomarker	Adjusted β (95% CI)	*p*
Temporal lobe involvement	beta‐syn	1.07 (0.48–1.66)	< 0.001
	Neurogranin	0.92 (0.37–1.48)	0.001
	SNAP‐25	1.08 (0.51–1.64)	< 0.001
	NfL	0.90 (0.28–1.52)	0.005
Multifocal lesions with grey and/or white matter involvement	beta‐syn	0.92 (0.09–1.74)	0.029

*Note:* β coefficients and 95% CIs from multivariable linear regression models of *z*‐scored biomarker levels. Each MRI pattern was modelled separately, adjusted for age, sex, time to LP and aetiology (HSV vs. other aetiologies). Only significant associations are shown; complete results are reported in Table S5.

Abbreviations: beta‐syn, beta‐synuclein; CI, confidence interval; HSV, herpes simplex virus; LP, lumbar puncture; NfL, neurofilament light chain protein; SNAP‐25, synaptosomal‐associated protein 25.

Multifocal lesions were associated with higher beta‐syn levels (*β* = 0.92, 95% CI 0.09–1.74; *p* = 0.029), whereas no significant associations were observed for the other biomarkers (Table 2). No independent associations were identified for basal ganglia/thalamus and/or brainstem involvement, non‐temporal lobe involvement or meningeal contrast enhancement (Table S5).

These results are summarised in the heatmap (Figure 2), which provides a standardised representation of the direction and magnitude of adjusted associations across MRI patterns and biomarkers. To facilitate clinical interpretation, model‐derived predicted biomarker values across MRI patterns are reported in Table S7. These findings are also illustrated in the radar plots (Figure 3; Figure S1).

**FIGURE 2:**
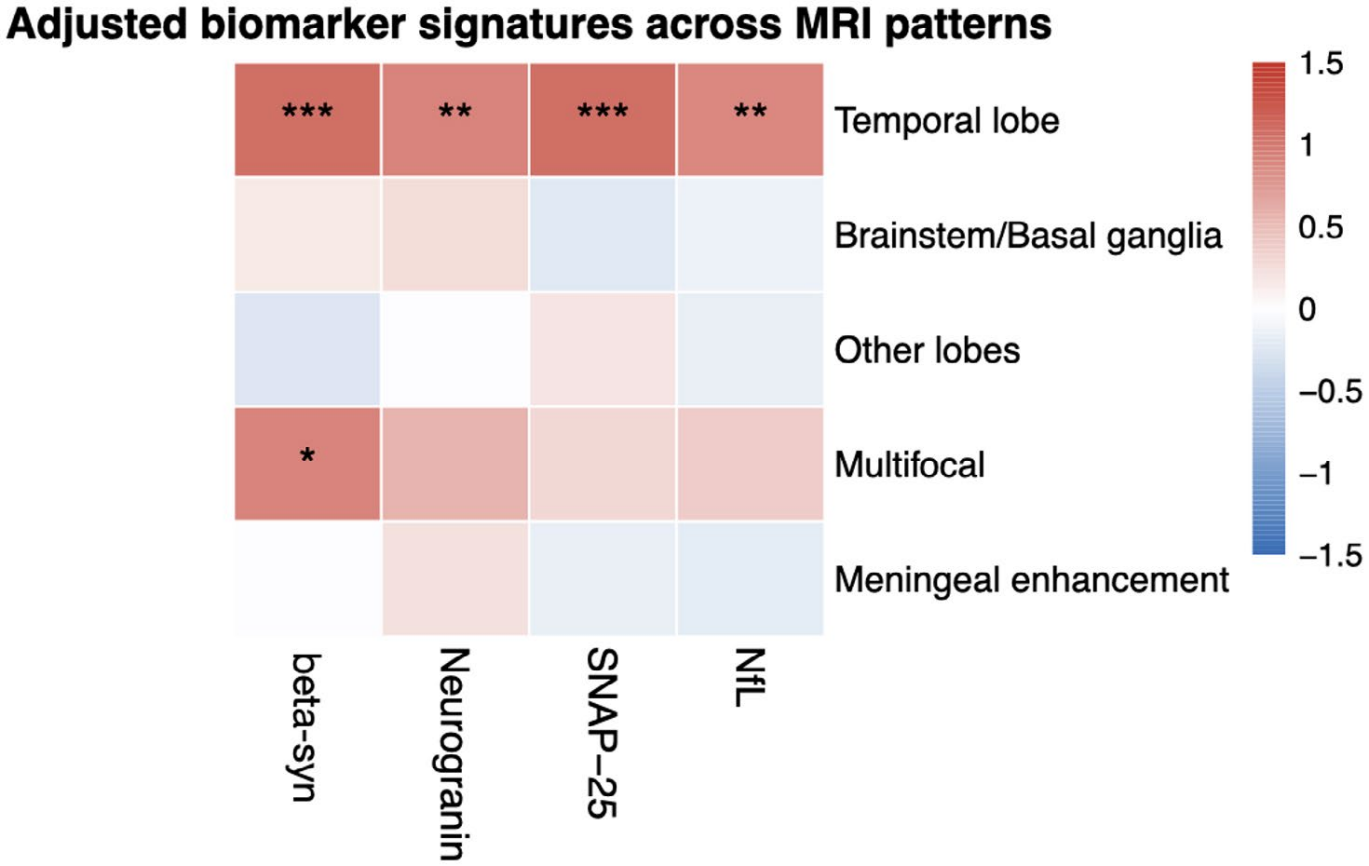
Adjusted biomarker signatures across MRI patterns. Heatmap of standardised regression coefficients (β) from multivariable linear regression models of *z*‐scored biomarker levels, adjusted for age, sex, time to LP and aetiology (HSV vs. other aetiologies). Warmer colours indicate higher biomarker levels and cooler colours indicate lower levels. Asterisks denote statistical significance (*p < 0.05; **p < 0.01; ***p < 0.001). Beta‐syn, beta‐synuclein; LP, lumbar puncture; NfL, neurofilament light chain protein; SNAP‐25, synaptosomal‐associated protein 25.

**FIGURE 3:**
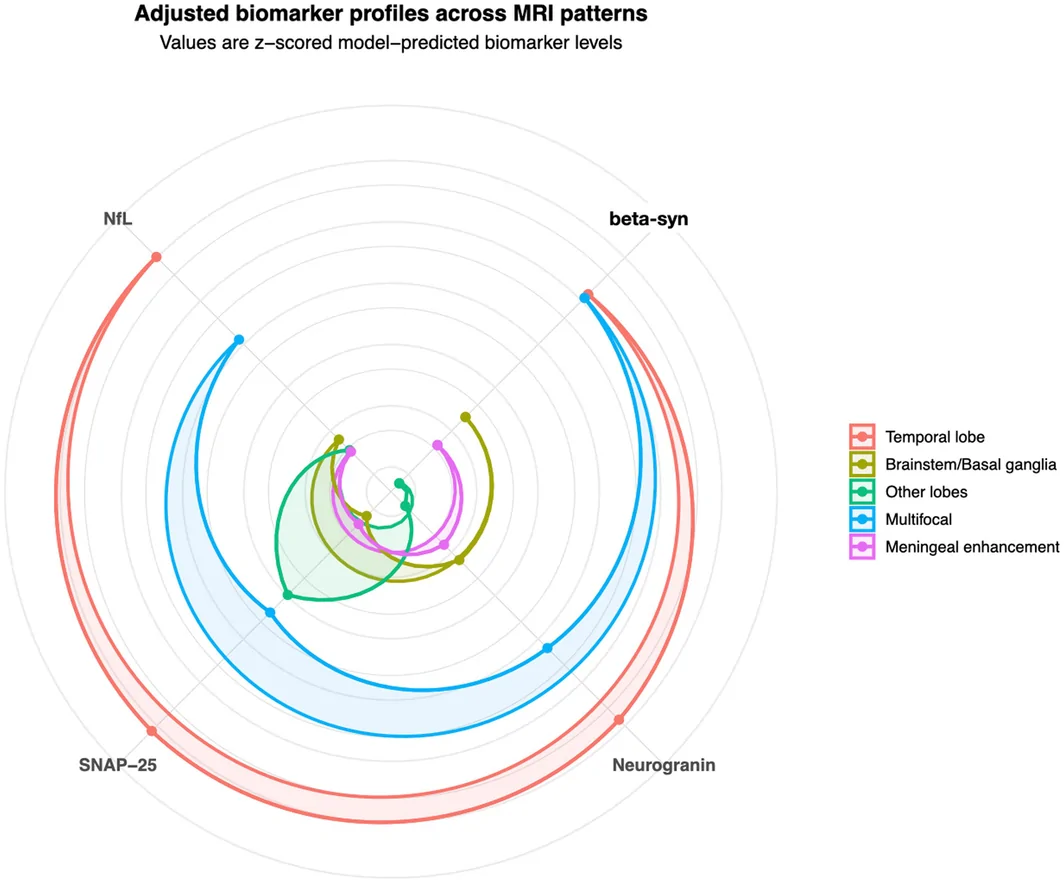
Adjusted biomarker profiles across MRI patterns. Radar plot of standardised (*z*‐scored) CSF biomarker levels predicted by multivariable linear regression models adjusted for age, sex, time to LP and aetiology (HSV vs. other aetiologies). Each axis represents one CSF biomarker (beta‐synuclein, neurogranin, SNAP‐25 and NfL). Temporal lobe involvement shows a distinct profile with globally elevated biomarker levels , while other MRI patterns display weaker and less consistent signatures, highlighting a differential biological burden across distinct imaging topographies. Beta‐syn, beta‐synuclein; HSV, herpes simplex virus; LP, lumbar puncture; NfL, neurofilament light chain protein; SNAP‐25, synaptosomal‐associated protein 25.

In exploratory ROC analyses, PC1, reflecting the global neuronal biomarker burden of synaptic and neuroaxonal injury, showed good discriminatory performance for temporal lobe involvement (AUC 0.796), which remained comparable after adjustment for demographic and clinical factors (AUC 0.777; DeLong *p* = 0.379) (Figure S2). In contrast, the discriminatory performance of PC1 for other MRI patterns was more modest and heterogeneous (Supplementary results).

### 3.3 Associations Between MRI Lesion Patterns, CSF Biomarker Profiles and Study Outcomes

Multivariable analyses evaluating the association between MRI features and clinical outcomes at discharge, after adjustment for age, sex, time to lumbar puncture, diagnostic aetiology and clinical severity at baseline (mRS at the time of CSF sampling), are reported in Table S8. Temporal lobe involvement was the only MRI pattern associated with poor functional outcome (mRS 3–6; OR 5.01, 95% CI 1.85–13.86; *p* = 0.002) and incomplete recovery (OR 4.65, 95% CI 1.72–12.60; *p* = 0.002), whereas no other MRI feature was significantly associated with either outcome.

PC1 explained 73.5% of the total variance and showed equal positive loadings for both variables, indicating that it captures a global neuronal biomarker burden reflecting combined synaptic and neuroaxonal damage. PC1 values were significantly higher in patients with temporal lobe involvement (*p* < 0.001) and in those with poor mRS (*p* = 0.008) and incomplete recovery (*p* = 0.013) at discharge (Figure 4). In multivariable logistic regression analysis for poor functional outcome at discharge (Table S9), temporal lobe involvement was associated with increased odds of poor mRS (OR 5.31, 95% CI 1.95–14.78; *p* = 0.001).

**FIGURE 4:**
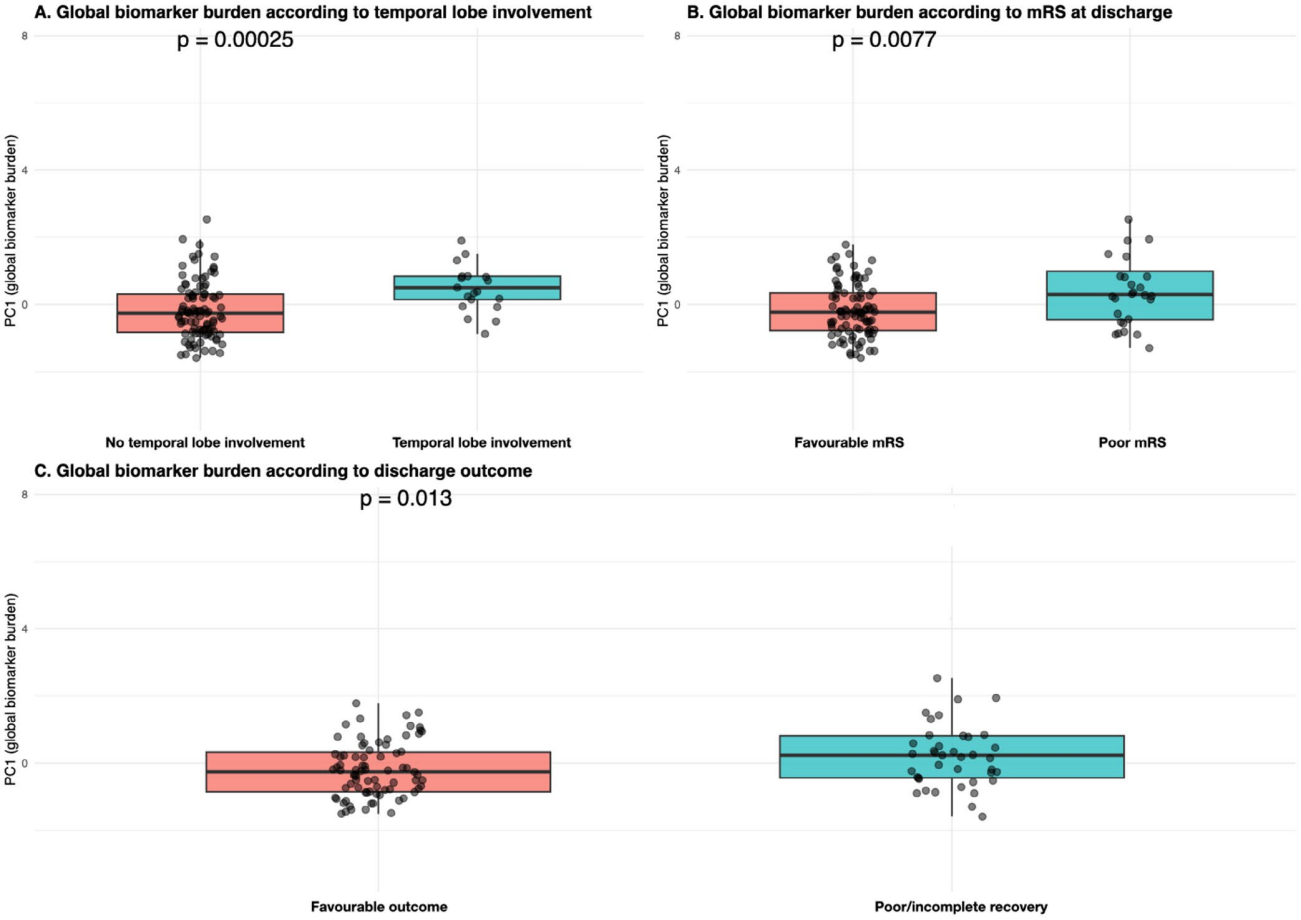
Global neuronal biomarker burden across MRI features and clinical outcomes. Distribution of PC1, reflecting the combined burden of synaptic and neuroaxonal injury, according to (A) temporal lobe involvement, (B) mRS at discharge (favourable mRS vs. poor mRS) and (C) outcome at discharge (favourable outcome/complete recovery vs. poor/incomplete recovery). Box plots indicate medians and interquartile ranges and dots represent individual patients. PC1 was higher in patients with temporal lobe involvement (*p* = 0.00025), poor mRS (*p* = 0.0077) and incomplete recovery (*p* = 0.013) (Wilcoxon rank‐sum test). mRS, modified Rankin Scale; PC1, first principal component derived from principal component analysis of the synaptic composite score (beta‐synuclein, neurogranin and SNAP‐25) and log‐transformed neurofilament light chain protein.

When PC1 was added to the model, both temporal lobe involvement and PC1 remained independently associated with outcome. In this combined model, the effect of temporal lobe involvement reflects its association with functional outcome after accounting for biomarker burden (OR 3.30, 95% CI 1.11–9.79; *p* = 0.030), whereas PC1 captures the effect of global biological injury independent of lesion location (OR 1.80, 95% CI 1.14–3.07; *p* = 0.022). The inclusion of PC1 significantly improved model fit (likelihood ratio test *p* = 0.010; AIC 119.2 vs. 123.9). A similar pattern was observed for incomplete recovery at discharge (Table S9). Temporal lobe involvement was associated with a higher likelihood of poor outcome in the base model (OR 4.12, 95% CI 1.57–11.45; *p* = 0.005). After inclusion of PC1, the association of temporal lobe involvement was attenuated (OR 2.78, 95% CI 0.98–8.15; *p* = 0.056), while PC1 remained independently associated with outcome (OR 1.58, 95% CI 1.05–2.51; *p* = 0.041). Model fit was significantly improved by the inclusion of PC1 (likelihood ratio test *p* = 0.026; AIC 146.9 vs. 149.8).

## 4 Discussion

The present multicentre study examined how MRI lesion patterns interact with CSF markers of synaptic and neuroaxonal injury and with short‐term clinical outcome across an aetiologically diverse ND population. Our findings demonstrate that the presence of MRI abnormalities is associated with an elevation of all investigated CSF biomarkers and that temporal lobe involvement represents the single MRI lesion pattern most consistently and independently associated with both higher biomarker concentrations and poor clinical outcome. Furthermore, adding a global neuronal biomarker score derived from principal component analysis to MRI topography significantly improved the fit of models for short‐term outcome.

The differences in CSF biomarker levels between controls, ND‐MRI− and ND‐MRI+ patients, more pronounced for beta‐syn and NfL and to a lesser extent for neurogranin and SNAP‐25, are consistent with the notion that structural brain involvement is associated with a higher degree of synaptic and neuroaxonal injury detectable in the CSF. This group‐dependent pattern is in accordance with previous observations from our group and others, showing that biofluid markers of neuroaxonal and synaptic damage are more elevated in ND cases with parenchymal involvement (i.e., [meningo‐]encephalitis) and more severe clinical presentation [8, 9, 10, 17, 18, 19]. Moreover, our study supports the hypothesis that MRI‐defined structural involvement is an independent driver of CSF biomarker elevation beyond clinical severity alone, a finding consistent with observations in other neuroinflammatory diseases, such as autoimmune encephalitis and multiple sclerosis, in which patients with MRI parenchymal abnormalities had higher NfL levels compared with those without [20, 21]. Notably, higher CSF neuronal biomarker concentrations in MRI‐positive patients were also observed when the analysis was restricted to patients with (meningo‐)encephalitis, suggesting that biomarker differences were not solely driven by the inclusion of meningitis and other non‐encephalitic presentations in the MRI‐negative group.

Among the MRI patterns examined, temporal lobe involvement was the feature most consistently associated with elevated CSF biomarker levels, even after multivariable adjustment and with an approximately five‐fold increase in the odds of poor functional outcome and incomplete recovery at discharge. Since HSV is the prototypical cause of temporal lobe involvement [3, 5], the observed associations might simply reflect HSV aetiology. However, temporal lobe involvement remained associated with higher biomarker levels after adjustment for HSV versus other aetiologies and patients with temporal involvement due to non‐HSV aetiologies showed biomarker levels comparable to those of HSV patients with temporal involvement. Analyses restricted to non‐HSV patients further supported this association, despite reduced statistical power. Thus, the association between temporal lobe involvement and neuronal and synaptic injury does not appear to be explained by HSV aetiology alone. The lack of consistent associations between biomarkers and other MRI patterns supports the hypothesis that lesion topography, rather than simply the presence of MRI abnormalities, may contribute to the neurochemical profile and short‐term outcome in NDs. Nevertheless, our findings should be interpreted cautiously. The relatively small number of patients with temporal lobe involvement and the close relationship between lesion topography, aetiology and clinical phenotype limit our ability to disentangle a topography‐specific effect from the underlying disease process. Residual confounding by disease severity also cannot be excluded.

Crucially, when a global neuronal biomarker score derived from PCA was added to the outcome model alongside temporal lobe involvement, both predictors remained independently associated with outcome and model fit was significantly improved, indicating that MRI lesion pattern and CSF biomarker burden capture distinct and complementary dimensions of disease severity.

This interaction between CSF biomarkers, temporal lobe involvement and short‐term outcome in NDs is biologically plausible for several reasons. First, the temporal lobe and its limbic structures are enriched in large calibre axons and show particularly high expression of synaptic proteins including neurogranin and beta‐syn and are particularly vulnerable to neurotropic pathogens [14, 22, 23, 24]. Moreover, temporal lobe damage is a common substrate for post‐infectious epileptogenesis, which may contribute substantially to neurological sequelae [25]. Finally, injury to the mesial temporal lobe, a critical hub for memory encoding, could result in persistent cognitive deficits that are major determinants of functional outcome after encephalitis [26]. As the prototypical example, HSV encephalitis, which characteristically involves the temporal lobe, is well known to be associated with extensive synaptic and neuroaxonal damage and temporal lobe signal abnormalities on MRI have been independently associated with poor outcome in this condition [3, 27]. Notably, in Alzheimer's disease, NfL and beta‐syn levels have also been associated with temporal lobe atrophy [23, 28] and combining MRI‐based temporal lobe involvement and biomarker data provides a more complete prognostic assessment than either parameter alone [29]. This suggests that the MRI pattern‐biomarker‐outcome interaction may extend beyond NDs and might be even a common finding in several neurological diseases. Further studies on NDs and other neurological disorders are needed to validate our results.

The present study has several strengths. First, it systematically integrates MRI lesion patterns and multiple CSF biomarkers in a well‐characterised multicentre cohort spanning diverse ND aetiologies, moving beyond single‐pathogen or single‐biomarker approaches. Second, the use of a pre‐specified, literature‐derived MRI classification framework ensured methodological consistency across centres. Third, the application of PCA to combine synaptic and neuroaxonal markers into a single biologically meaningful composite represents a methodologically robust approach to reducing dimensionality while preserving biological information.

However, several limitations must be acknowledged. First, the retrospective and cross‐sectional design, small sample sizes within individual ND subgroups and the relatively high proportion of temporal lobe involvement among MRI+ patients may limit both the statistical power for aetiology‐specific analyses and the generalisability of our findings to cases with lesions in other brain regions. Moreover, outcome was assessed only at discharge, without long‐term follow‐up data, which precludes conclusions on long‐term outcome. In addition, given the limited number of outcome events, the multivariable models may be overfitted and their predictive performance requires internal and external validation. Furthermore, the MRI classification was performed by three study investigators based on official neuroradiology reports and a pre‐specified taxonomy, rather than by dedicated neuroradiologists with formal inter‐rater reliability assessment, thus representing a potential source of classification bias. MRI was not performed at standardised time points and in some patients early imaging may have missed evolving parenchymal lesions, potentially leading to misclassification of MRI‐negative cases; however, such misclassification would be expected to reduce rather than increase the observed biomarker differences between MRI‐positive and MRI‐negative patients. Finally, neuroinflammatory biomarkers (e.g., glial fibrillary acidic protein, GFAP) [10] were not assessed in this cohort and will be addressed in future studies.

In conclusion, MRI topography and CSF biomarkers of synaptic and neuroaxonal damage provide complementary information in NDs. Temporal lobe involvement and a global neuronal biomarker score were independently associated with short‐term outcome. Whether their combined assessment can support early risk stratification and prognostic assessment needs to be established in prospective studies with larger cohorts, independent validation and long‐term follow‐up.

## Author Contributions

**Lucio D'Anna:** conceptualization, data curation, investigation, formal analysis, writing – review and editing, writing – original draft, software, methodology, validation, visualisation, supervision, project administration. **Deborah K. Erhart:** investigation, data curation, writing – review and editing. **Franz Felix Konen:** data curation, investigation, writing – review and editing. **Christopher M. Weise:** data curation, investigation, visualisation, writing – review and editing. **Makbule Senel:** investigation, writing – review and editing. **Caroline Stapf:** investigation, data curation, writing – review and editing. **Dominica Hudasch:** data curation, investigation, writing – review and editing. **Petra Steinacker:** methodology, investigation, resources, writing – review and editing. **Lorenzo Barba:** data curation, formal analysis, writing – review and editing, investigation. **Patrick Oeckl:** methodology, validation, investigation, writing – review and editing. **Jonas Ranke:** data curation, investigation, writing – review and editing. **Nicola Ticozzi:** resources, investigation, writing – review and editing. **Federico Verde:** resources, investigation, writing – review and editing. **Steffen Halbgebauer:** methodology, validation, writing – review and editing. **Thomas Skripuletz:** writing – review and editing, supervision, resources, data curation, investigation. **Kurt‐Wolfram Sühs:** data curation, investigation, resources, writing – review and editing, supervision. **Hayrettin Tumani:** data curation, supervision, resources, writing – review and editing, investigation. **Markus Otto:** data curation, supervision, resources, conceptualization, writing – review and editing, investigation, funding acquisition. **Samir Abu‐Rumeileh:** conceptualization, project administration, software, investigation, methodology, data curation, supervision, formal analysis, writing – review and editing, writing – original draft, visualisation, validation.

## Funding

The Medical Faculty of Martin‐Luther‐University Halle‐Wittenberg supported CS (Clinician Scientist Program No. CS25/01), LB (Junior Clinician Scientist Program No. JCS24/02), JR (Junior Clinician Scientist Program No. JCS25/04), SAR (Clinician Scientist Program No. CS22/06), and, through its Roux Program, MO; CS was additionally supported by the Doris Ruess Stiftung. FFK is a fellow of the PRACTIS Clinician Scientist Program funded by the DFG and Hannover Medical School and has been further supported by the Erwin Röver Foundation. KWS acknowledges DFG funding under Germany's Excellence Strategy (EXC 2155, project number 390874280). CMW has been supported by Novartis (MOMB157A_FVMS001), outside the present study. Grants to PO comprise ALS Association/ALS Finding A Cure (24‐SGP‐691, 23‐PPG‐674‐2), Charcot Foundation (D.7090), Cure Alzheimer's Fund, DZNE Innovation‐to‐Application (I2A_call9_Oeckl) and EU Horizon Europe (SYNAPSING). MO acknowledges the German Federal Ministry of Education and Research (FTLDc, 01GI1007A), the EU Moodmarker program (01EW2008), EU‐MIRIADE, the ALS Association, the foundation of the state Baden‐Württemberg (D.3830), Boehringer Ingelheim Ulm University Biocentre (D.5009) and the Thierry Latran Foundation. Institutional research funding to HT was provided by the Ministry of Science and Arts of the State of Baden‐Württemberg, the Bundesministerium für Gesundheit (BMG), the Deutsche Multiple Sklerose Gesellschaft (DMSG) and Chemische Fabrik Karl Bucher GmbH. FV has been supported by the Italian Ministry of Health and by the Fondazione Regionale per la Ricerca Biomedica (FRRB) of Regione Lombardia. NT and FV further acknowledge grants from the Italian Ministry of Health (Ricerca Finalizzata RF‐2021‐12,374,238 ‘DRIVEALS’; Hub Life Science–Diagnostica Avanzata, HLS‐DA, PNC‐E3‐2022‐23,683,266, within the Complementary National Plan Innovative Health Ecosystem) and from the Italian Ministry of Education and Research (‘Dipartimenti di Eccellenza’ Program 2023–2027, Department of Pathophysiology and Transplantation, Università degli Studi di Milano). None of the funding bodies listed above had any influence on the design of the study, on data collection, analysis or interpretation, on the drafting of the manuscript or on the decision to submit it for publication.

## Ethics Statement

The study complied with Good Clinical Practice standards and with the Declaration of Helsinki in its revised form. The local Ethics Committees of the four participating centres granted approval: Ulm University Hospital (20/10), Hannover Medical School (7837_BO_K_2018), Martin‐Luther‐University Halle‐Wittenberg (2021–101) and IRCCS Istituto Auxologico Italiano, Milan (2021_05_18_04). Written informed consent was obtained from all participants included in the study or their legal guardians.

## Conflicts of Interest

Deborah K. Erhart has been granted travel support by Alexion, Argenx and Novartis, none of which relates to the present study. Franz Felix Konen reports travel support and lecture honoraria from Argenx, Alexion, Novartis, Merck and Takeda, together with research funding from Merck and Siemens, all outside the present study. Christopher M. Weise has been remunerated by Eli Lilly for invited lectures, outside the present work. Makbule Senel reports honoraria for consultancy and/or lectures from Amgen/Horizon, Alexion, Biotest, Bayer, Biogen, Bristol‐Myers‐Squibb/Celgene, Janssen, Merck, Roche, Sanofi Genzyme and UCB, with no relation to the submitted work. Thomas Skripuletz reports funding for research from Alnylam, CSL Behring, Merck, Novartis and Siemens, as well as fees for consultancy and/or lectures from Amgen, Alexion, Alnylam, argenx, Biogen, Bayer, BMS, Centogene, CSL Behring, Grifols, Hexal, Horizon, Janssen, Merck, Novartis, Pfizer, Purpose pharma, Roche, Sanofi, Siemens, SOBI, Takeda, Teva and Viatris, none of which bear on the present study. Kurt‐Wolfram Sühs has been remunerated for lectures or reimbursed for meeting attendance by Bavarian Nordic, Biogen, Bristol‐Myers, Merck, Mylan, Novartis, Roche, Viatris and Bristol‐Myers Squibb and has received research funding from Bristol‐Myers Squibb, unconnected with the submitted work. Hayrettin Tumani has been remunerated for consultancy or speaking activities and/or for participation in events supported by Alexion, Bayer, Biogen, Bristol‐Myers Squibb, Celgene, Diamed, Fresenius, Fujirebio, GlaxoSmithKline, Hexal, Horizon, Janssen‐Cilag, Merck, Novartis, Ottobock, Roche, Sanofi‐Genzyme, Siemens, Teva, UCB and Viatris. Patrick Oeckl reports fees for consultancy from Fundamental Pharma and LifeArc, lecture honoraria from GSK and Fujirebio and travel support from Biogen. Nicola Ticozzi has been remunerated for consultancy and/or speaking activities by Amylyx Pharmaceutical, Biogen, Italfarmaco and Zambon Biotech SA, outside the submitted work. Markus Otto reports fees for consultancy from Axon, Biogen, Grifols and Roche and serves on the ATLAS trial board of Biogen, neither of which relates to the work presented here. With respect to non‐financial interests, Nicola Ticozzi serves as associate editor of Frontiers in Aging Neuroscience and on the advisory board of Neurological Sciences, Federico Verde as Associate Editor of Journal of Alzheimer's Disease, Samir Abu‐Rumeileh as member of the editorial board of Scientific Reports and Markus Otto as speaker of the German FTLD consortium. A patent application covering the measurement of beta‐synuclein in blood has been filed with Markus Otto, Steffen Halbgebauer and Patrick Oeckl as co‐inventors. No competing interests are declared by the remaining authors.

## Supporting information


**Table S1:** Demographic characteristics and biomarker distribution of the diagnostic groups.
**Table S2:** Demographic characteristics and results of diagnostic investigations of ND cases.
**Table S3:** Sensitivity analysis restricted to patients with (meningo‐)encephalitis.
**Table S4:** CSF biomarker concentrations according to temporal lobe involvement and HSV aetiology.
**Table S5:** Adjusted associations between MRI patterns and CSF biomarkers.
**Table S6:** Multivariable linear regression models in non‐HSV patients (*n* = 103).
**Table S7:** Model‐predicted adjusted CSF biomarker values across MRI patterns.
**Table S8:** Multivariable associations between MRI features and clinical outcomes adjusted for age, sex, time to lumbar puncture, diagnostic aetiology and mRS at the time of the lumbar puncture.
**Table S9:** Regression models for short‐term outcomes according to temporal lobe involvement and global biomarker burden.
**Figure S1:** Adjusted biomarker profiles for individual MRI lesion patterns. Radar plots of standardised (z‐scored) CSF biomarker levels predicted by multivariable linear regression models adjusted for age, sex, time to LP and aetiology (HSV vs. other aetiologies). Each panel represents one MRI pattern and each axis represents one biomarker (beta‐synuclein, neurogranin, SNAP‐25 and NfL). beta‐syn, beta‐synuclein; HSV, herpes simplex virus; LP, lumbar puncture; NfL, neurofilament light chain protein; SNAP‐25, synaptosomal‐associated protein 25.
**Figure S2:** Discriminatory performance of PC1 for temporal lobe involvement.
ROC curves of PC1 for the discrimination of temporal lobe involvement, unadjusted (AUC 0.796) and adjusted for age, sex and time to LP (AUC 0.777; DeLong test *p* = 0.379). The diagonal line indicates chance‐level discrimination. AUC, area under the curve; LP, lumbar puncture; PC1, first principal component reflecting the combined burden of synaptic and neuroaxonal injury; ROC, receiver operating characteristic.

## Data Availability

The data that support the findings of this study are available on request from the corresponding author. The data are not publicly available due to privacy or ethical restrictions.
