# Supplementary material for: Convergence of Cerebrospinal Fluid Neuronal Biomarker Burden and MRI Lesion Pattern and Its Association With Short‐Term Outcome in Neuroinfectious Diseases
Source: Eur J Neurol. 2026 Nov 2;33(11):e70802. doi: 10.1111/ene.70802 (PMC13725939; doi:10.1111/ene.70802)
Supplement: Supplementary file 1 [file ENE-33-e70802-s001.docx]

**SUPPORTING INFORMATION**

**SUPPLEMENTARY METHODS**

*Study design and participants*

Diagnostic group distributions among centres were the following: Halle cohort: 14 ND, 42 controls; Ulm cohort: 85 ND, 15 controls; Hannover cohort: 24 ND, 18 controls; Milan cohort: 20 controls.

Pathogen identification (viral or bacterial) was achieved in 94 patients through polymerase chain reaction (PCR), culture or microscopic examination, or determination of pathogen-specific intrathecal antibody synthesis via antibody index (AI) calculation in paired CSF/serum samples. The ND group included the following etiologies: herpes simplex virus type 1 and 2 (HSV; n=20), varicella-zoster virus (VZV; n=21), tick-borne encephalitis (TBE; n=16), bacterial meningitis/(meningo-)encephalitis (BM; n=21), Lyme neuroborreliosis (LNB; n=14; all definite cases according to Stanek et al.[1]), COVID-19 infection (n=1), bacterial abscess (n=1), and infectious neurological disease of unknown or undetermined aetiology (n=29). In the latter subgroup, a viral or atypical pathogen etiology was considered most likely based on clinical presentation (meningitis, encephalitis, myelitis, radiculitis or cranial nerve palsies) and CSF findings (white blood cell count >5/µL), despite negative routine pathogen investigations [2]. The majority of patients (n=95) had not yet commenced antiviral or antibiotic treatment at the time of LP.

Non-inflammatory controls included individuals with primary headache disorders, subjective cognitive decline, psychiatric conditions, sleep disorders, or non-inflammatory polyneuropathies.

*Clinical, neuroimaging and biomarker data*

CSF samples were collected by LP, centrifuged when blood contamination was present, aliquoted, and stored at −80°C in polypropylene tubes until analysis at each participating centre. Beta-synuclein (beta-syn) was quantified using a previously validated in-house immunoassay [3,4]. SNAP-25 was measured using a commercial single molecule array (Simoa) kit (Quanterix, Lexington, MA, USA), and neurogranin using a commercial immunoassay (Euroimmun, Lübeck, Germany) [4]. NfL was measured using a commercially available kit on the ELLA microfluidic platform (Bio-Techne, Minneapolis, MN, USA). All measurements were performed in a randomised order and analysts were blinded to sample identity and clinical diagnosis. Intra-assay and inter-assay coefficients of variation were <10% and <15%, respectively, for all biomarker assays [4].

**Results**

*Associations between MRI patterns and CSF biomarker profiles and their discriminatory performance*

For brainstem and basal ganglia involvement, discrimination improved after adjustment (AUC 0.80 vs 0.69), although this difference was not statistically significant (p=0.243), suggesting a partial contribution of clinical covariates. A similar pattern was observed for multifocal lesions, where adjustment significantly improved discrimination (AUC 0.78 vs 0.64; DeLong p=0.041), indicating that the combination of biomarker burden and clinical factors may enhance detection in this subgroup. Conversely, PC1 showed limited discriminatory ability with regard to the involvement of other brain lobes (AUCs <0.70), and adjustment did not improve model performance.

**Supplementary Table 1. Demographic characteristics and biomarker distribution of the diagnostic groups.**

|  | **Controls** | **ND-MRI^-^** | **ND-MRI^+^** | **p value** |
| --- | --- | --- | --- | --- |
| **N** | 95 | 64 | 59 |  |
| **Age (y), median (IQR)** | 52.5 (38-68) | 49.9 (36-64) | 65 (50-74.3) | 0.001 |
| **Sex, Female n (%)** | 54 (57%) | 29 (45%) | 24 (41%) | 0.115 |
| **CSF beta-syn (pg/ml) median (IQR)** | 187 (127-239) | 219 (147.3-307.5) | 314 (222.2-499.4) | <0.001 |
| **CSF SNAP-25 (pg/ml) median (IQR)** | 47.9 (37.1-63.9) | 42.9 (27.9-61.8) | 54.1 (41.3-72.7) | 0.014 |
| **CSF neurogranin (pg/ml) median (IQR)** | 195 (126-258) | 215.8 (110-320) | 268. (159-499.6) | 0.002 |
| **CSF NfL (pg/ml) median (IQR)** | 740 (466-1349) | 1126.5 (517.2-3565.2) | 3572 (1563-16649) | <0.001 |

Values are median (IQR) unless otherwise specified. p values: Kruskal–Wallis test for continuous variables and χ² test for categorical variables.
Abbreviations: beta-syn, beta-synuclein; IQR, interquartile range; ND-MRI−/ND-MRI+, patients with neuroinfectious diseases without/with MRI abnormalities; NfL, neurofilament light chain protein; SNAP-25, synaptosomal-associated protein 25.

**Supplementary Table 2. Demographic characteristics and results of diagnostic investigations of ND cases.**

| **Diagnostic group** | **ND** |
| --- | --- |
| **N** | 123 |
| **Age (years) mean±SD** | 55.23 ± 18.04 |
| **Female (%)** | 43.1 |
| **Aetiologies N** | 21 VZV, 20 HSV, 16 TBE, 21 BM, 14 LNB, 31 other/unknown |
| **Clinical picture** N(%) | (meningo-)encephalitis: 85 (69.1), meningitis: 32 (26.0), other: 6 (4.9) |
| **Disease duration at LP (days)** Mean ± SD | 10.4± 9.6 |
| **Presenting symptoms/signs** |  |
| Altered mental status/consciousness N(%) | 35 (28.5) |
| Cognitive deficits N(%) | 44 (35.8) |
| Psychiatric symptoms N(%) | 15 (12.2) |
| Seizures N(%) | 27 (22.0) |
| Fever N(%) | 60 (48.8) |
| Headache N(%) | 91 (74.0) |
| Meningism N(%) | 23 (18.7) |
| Focal neurologic signs N(%) | 17 (13.8) |
| Peripheral nervous system signs/symptoms N(%) | 14 (11.4) |
| Autonomic dysfunction N(%) | 9 (7.3) |
| **Clinical course** |  |
| Requirement for ICU admission N(%) | 25 (20.3) |
| Duration of hospitalisation (days) Mean ± SD | 17.22 ± 13.17 |
| **Brain /spinal MRI:**  **normal N(%)**  **abnormal N(%)**^†^  **Involvement:**  Temporal involvement (uni and/or bilateral)  Other lobe involvement (unilateral and/or bilateral)  basal ganglia /thalamus  brainstem  cranial and/or peripheral nerves  spinal cord  **Pattern:**  multifocal with white matter involvement  multifocal with grey and white matter involvement  space occupying and/or ring-enhancing lesion  meningeal contrast enhancement  extra-axial lesion | 64 (52.0)  59 (48.0)  21 (17.1)  9 (7.3)  4 (3.2)  5 (4.1)  2 (1.6)  4 (3.2)  8 (6.5)  10 (8.1)  6 (4.9)  13 (10.6)  4 (3.2) |
| **CSF: pleocytosis,**  **positive oligoclonal bands** N(%) | 123 (100);  45 (36.6) |
| **EEG: abnormal N(%)**^‡^ | 33 (57.9) |
| **Interval symptom onset–MRI (days),** mean ± SD  All ND  ND MRI-  ND MRI+ | 10.7 ± 8.5  8.9 ± 6.8  12.8 ± 10.1 |
| **Under treatment at time of LP** N(%) | 23 (18.7) |
| **mRS at LP:**  0-2 N(%)  3-6 N(%) | 77 (62.6)  46 (37.4) |
| **Outcome at discharge:** ^§^  Full recovery N(%)  Remaining deficits or death N(%) | 79 (65.3)  42 (34.7) |
| **mRS at discharge:** ^§^  0-2 N(%)  3-6 N(%) | 92 (76.0)  29 (24.0) |

†The sum of locations and patterns exceeds the total number of MRI+ patients (N=59) as some patients presented with lesions in multiple regions and/or more patterns simultaneously, ‡available in 57 ND, §available for 121 ND,

Abbreviations: BM, bacterial meningitis/meningoencephalitis; EEG, electroencephalogram; HSV, herpes simplex virus; ICU, intensive care unit; LNB, Lyme neuroborreliosis; LP, lumbar puncture; mRS, modified Rankin Scale; ND, neuroinfectious diseases; SD, standard deviation; TBE, tick-borne encephalitis; VZV, varicella-zoster virus.

**Supplementary Table 3. Sensitivity analysis restricted to patients with (meningo-)encephalitis**

| **Biomarker** | **MRI−** | **MRI+** | **p value** |
| --- | --- | --- | --- |
| beta-syn | 256.0 (141.2–327.5) | 346.2 (222.3–541.4) | 0.011 |
| Neurogranin | 222.0 (98.4–341.0) | 300.3 (179.8–511.2) | 0.017 |
| SNAP-25 | 47.9 (28.8–66.1) | 56.8 (43.5–79.0) | 0.016 |
| NfL | 2278 (899.2–5305.8) | 3821 (1699.5–17023) | 0.037 |

Values are median (interquartile range). The analysis was restricted to 85 patients with (meningo-)encephalitis (MRI−, n=38; MRI+, n=47), except for SNAP-25, for which one value was missing in the MRI+ group (n=46). P values were derived from Wilcoxon rank-sum tests.

Abbreviations: beta-syn, beta-synuclein; MRI, magnetic resonance imaging; NfL, neurofilament light chain; SNAP-25, synaptosomal-associated protein 25.

**Supplementary Table 4. CSF biomarker concentrations according to temporal lobe involvement and HSV aetiology**

| **Biomarker (pg/ml)** | **Temporal involvement,**  **HSV (n=11)** | **Temporal involvement,**  **non-HSV (n=10)** | **No temporal involvement,**  **non-HSV (n=93)** | **p (a)** | **p (b)** |
| --- | --- | --- | --- | --- | --- |
| beta-syn | 317.8 (219.6–988.7) | 469.3 (303.9–626.2) | 244.3 (173.0–328.3) | 0.549 | 0.002 |
| Neurogranin | 368.5 (289.5–603.5) | 497.1 (232.6–518.7) | 200.7 (123.9–316.5) | 1.000 | 0.006 |
| SNAP-25 | 74.9 (41.8–119.7) | 66.3 (60.1–70.9) | 45.1 (29.0–59.8)* | 0.751 | 0.004 |
| NfL | 2803 (2189–55665) | 7904 (3619–15668) | 1928 (780–5448) | 0.699 | 0.030 |

Values are median (interquartile range). Temporal involvement, non-HSV: bacterial meningoencephalitis, n=6; VZV, n=4. The nine HSV patients without temporal lobe involvement are not shown. (a) Temporal HSV vs temporal non-HSV; (b) temporal non-HSV vs non-temporal non-HSV. P values were derived from Wilcoxon rank-sum tests.

Abbreviations: beta-syn, beta-synuclein; HSV, herpes simplex virus; MRI, magnetic resonance imaging; NfL, neurofilament light chain; SNAP-25, synaptosomal-associated protein 25; VZV, varicella-zoster virus.

### **Supplementary Table 5. Adjusted associations between MRI patterns and CSF biomarkers.**

| **MRI pattern** | **Biomarker** | **Adjusted β (95% CI)** | **P value** |
| --- | --- | --- | --- |
| Temporal lobe involvement | beta-syn | 1.07 (0.48–1.66) | <0.001 |
| Temporal lobe involvement | Neurogranin | 0.92 (0.37–1.48) | 0.001 |
| Temporal lobe involvement | SNAP-25 | 1.08 (0.51–1.64) | <0.001 |
| Temporal lobe involvement | NfL | 0.90 (0.28–1.52) | 0.005 |
| Basal ganglia/ thalamus and/or brain stem involvement | beta-syn | 0.16 (-0.84–1.16) | 0.750 |
| Basal ganglia/ thalamus and/or brain stem involvement | Neurogranin | 0.25 (-0.67–1.18) | 0.588 |
| Basal ganglia/ thalamus and/or brain stem involvement | SNAP-25 | -0.22 (-1.18–0.75) | 0.658 |
| Basal ganglia/ thalamus and/or brain stem involvement | NfL | -0.12 (-1.16–0.91) | 0.817 |
| Other lobe involvement (non-temporal) | beta-syn | -0.25 (-1.12–0.63) | 0.575 |
| Other lobe involvement (non-temporal) | Neurogranin | -0.01 (-0.82–0.81) | 0.983 |
| Other lobe involvement (non-temporal) | SNAP-25 | 0.19 (-0.70–1.08) | 0.671 |
| Other lobe involvement (non-temporal) | NfL | -0.16 (-1.07–0.75) | 0.729 |
| Multifocal with grey and white matter involvement | beta-syn | 0.92 (0.09–1.74) | 0.029 |
| Multifocal with grey and white matter involvement | Neurogranin | 0.57 (-0.21–1.35) | 0.149 |
| Multifocal with grey and white matter involvement | SNAP-25 | 0.30 (-0.51–1.11) | 0.466 |
| Multifocal with grey and white matter involvement | NfL | 0.37 (-0.49–1.24) | 0.395 |
| Meningeal contrast enhancement | beta-syn | 0.00 (-0.75–0.74) | 0.995 |
| Meningeal contrast enhancement | Neurogranin | 0.22 (-0.47–0.91) | 0.536 |
| Meningeal contrast enhancement | SNAP-25 | -0.18 (-0.89–0.54) | 0.622 |
| Meningeal contrast enhancement | NfL | -0.18 (-0.95–0.59) | 0.639 |

β coefficients and 95% CIs from multivariable linear regression models of z-scored biomarker levels. Each MRI pattern was modelled separately, adjusted for age, sex, time to LP and aetiology (HSV vs other aetiologies).

Abbreviations: beta-syn, beta-synuclein; CI, confidence interval; HSV, herpes simplex virus; LP, lumbar puncture; NfL, neurofilament light chain protein; SNAP-25, synaptosomal-associated protein 25.

**Supplementary Table 6. Multivariable linear regression models in non-HSV patients (n=103).**

| **Biomarker** | **Adjusted β (95% CI)** | **p value** |
| --- | --- | --- |
| beta-syn | 0.77 (0.16 to 1.37) | 0.013 |
| Neurogranin | 0.83 (0.21 to 1.45) | 0.010 |
| SNAP-25 | 0.60 (−0.03 to 1.23) | 0.060 |
| NfL | 0.15 (−0.52 to 0.81) | 0.663 |

β coefficients and 95% CIs for temporal lobe involvement (n=10) vs no temporal lobe involvement (n=93) from multivariable linear regression models of z-scored biomarker levels in patients without HSV infection, adjusted for age, sex and time to LP.

Abbreviations: beta-syn, beta-synuclein; CI, confidence interval; HSV, herpes simplex virus; LP, lumbar puncture; NfL, neurofilament light chain protein; SNAP-25, synaptosomal-associated protein 25.

**Supplementary Table 7. Model-predicted adjusted CSF biomarker values across MRI patterns.**

| **MRI pattern** | **Predicted beta-syn (pg/ml)** | **Predicted Neurogranin (pg/ml)** | **Predicted SNAP-25 (pg/ml)** | **Predicted NfL (pg/ml)** |
| --- | --- | --- | --- | --- |
| Temporal lobe involvement | 809.3 | 453.0 | 89.9 | 64162.2 |
| Basal ganglia/ thalamus and/or brain stem involvement | 501.2 | 350.6 | 52.2 | 18379.6 |
| Other lobe involvement (non-temporal) | 334.8 | 315.9 | 66.0 | 15722.2 |
| Multifocal with grey and /or white matter involvement | 800.6 | 407.0 | 69.1 | 43433.0 |
| Meningeal contrast enhancement | 430.9 | 340.7 | 53.6 | 15356.1 |

Abbreviations: beta-syn, beta-synuclein; NfL, neurofilament light chain protein; SNAP-25, synaptosomal-associated protein 25.

## **Supplementary Table 8. Multivariable associations between MRI features and clinical outcomes adjusted for age, sex, time to lumbar puncture, diagnostic aetiology and mRS at the time of the lumbar puncture.**

| **MRI feature** | **Poor mRS at discharge**  **OR (95% CI)** | **p-value** | **Incomplete recovery at discharge OR (95% CI)** | **p-value** |
| --- | --- | --- | --- | --- |
| **Temporal lobe involvement** | 5.01 (1.85–13.86) | 0.002 | 4.65 (1.72–12.60) | 0.002 |
| **Basal ganglia/ thalamus and/or brain stem involvement** | 2.54 (0.48–12.23) | 0.242 | 1.38 (0.30–6.35) | 0.676 |
| **Other lobe involvement (non-temporal)** | 1.65 (0.33–6.74) | 0.498 | 3.04 (0.75–12.38) | 0.120 |
| **Multifocal with grey and/or white matter involvement** | 1.40 (0.29–5.44) | 0.642 | 0.78 (0.19–3.14) | 0.721 |
| **Meningeal contrast enhancement** | 1.48 (0.37–4.96) | 0.545 | 1.63 (0.52–5.10) | 0.402 |

Odds ratios and 95% CIs from multivariable logistic regression models; each MRI feature was modelled separately. Poor functional outcome was defined as mRS 3–6 at discharge.

Abbreviations: CI, confidence interval; mRS, modified Rankin Scale; OR, odds ratio.

**Supplementary Table 9. Regression models for short-term outcomes according to temporal lobe involvement and global biomarker burden**

| **Outcome** | **Model** | **Variable** | **OR (95% CI)** | **P value** |
| --- | --- | --- | --- | --- |
| Poor mRS at discharge | Base model | Temporal lobe involvement | 5.31 (1.95–14.78) | 0.001 |
| Poor mRS at discharge | Base model + PC1 | Temporal lobe involvement | 3.30 (1.11–9.79) | 0.030 |
| Poor mRS at discharge | Base model + PC1 | Global biomarker burden (PC1) | 1.80 (1.14–3.07) | 0.022 |
| Incomplete recovery at discharge | Base model | Temporal lobe involvement | 4.12 (1.57–11.45) | 0.005 |
| Incomplete recovery at discharge | Base model + PC1 | Temporal lobe involvement | 2.78 (0.98–8.15) | 0.056 |
| Incomplete recovery at discharge | Base model + PC1 | Global biomarker burden (PC1) | 1.58 (1.05–2.51) | 0.041 |

Likelihood ratio tests for adding PC1 were significant for both outcomes (poor mRS at discharge: p=0.010; incomplete recovery at discharge: p=0.026). Lower AIC values in the models including PC1 supported improved model fit. PC1 was derived from principal component analysis of the synaptic composite score and log-transformed NfL values. Base model includes temporal lobe involvement alone; Base model + PC1 includes temporal lobe involvement and PC1. Models adjusted for age, sex, time to lumbar puncture, diagnostic aetiology, and baseline clinical severity (mRS at the time of CSF sampling).

Abbreviations: AIC, Akaike information criterion; CI, confidence interval; HSV, herpes simplex virus; LP, lumbar puncture; mRS, modified Rankin Scale; OR, odds ratio; PC1, first principal component reflecting the combined burden of synaptic and neuroaxonal injury.

**Supplementary Figure 1. Adjusted biomarker profiles for individual MRI lesion patterns.** Radar plots of standardised (z-scored) CSF biomarker levels predicted by multivariable linear regression models adjusted for age, sex, time to LP and aetiology (HSV vs other aetiologies). Each panel represents one MRI pattern, and each axis one biomarker (beta-synuclein, neurogranin, SNAP-25 and NfL)
Abbreviations: beta-syn, beta-synuclein; HSV, herpes simplex virus; LP, lumbar puncture; NfL, neurofilament light chain protein; SNAP-25, synaptosomal-associated protein 25.

**Supplementary Figure 2. Discriminatory performance of PC1 for temporal lobe involvement.**
ROC curves of PC1 for the discrimination of temporal lobe involvement, unadjusted (AUC 0.796) and adjusted for age, sex and time to LP (AUC 0.777; DeLong test p=0.379). The diagonal line indicates chance-level discrimination.
Abbreviations: AUC, area under the curve; LP, lumbar puncture; PC1, first principal component reflecting the combined burden of synaptic and neuroaxonal injury; ROC, receiver operating characteristic.

**References**

1. Stanek G, Fingerle V, Hunfeld KP, et al. Lyme borreliosis: clinical case definitions for diagnosis and management in Europe. Clin Microbiol Infect. 2011;17:69-79. doi:10.1111/j.1469-0691.2010.03175.x
2. Erhart DK, Klose V, Schäper T, Tumani H, Senel M. CXCL13 in cerebrospinal fluid: clinical value in a large cross-sectional study. Int J Mol Sci. 2023;25:425. doi:10.3390/ijms25010425
3. Abu-Rumeileh S, Halbgebauer S, Bentivenga GM, et al. High diagnostic performance of plasma and cerebrospinal fluid beta-synuclein for sporadic Creutzfeldt-Jakob disease. Ann Clin Transl Neurol. 2023;10:1904-1909. doi:10.1002/acn3.51873
4. Abu-Rumeileh S, Erhart DK, Barba L, et al. CSF beta-synuclein, SNAP-25, and neurogranin in infectious and autoimmune inflammatory neurologic diseases. Neurol Neuroimmunol Neuroinflamm. 2025;12:e200491. doi:10.1212/NXI.0000000000200491
